# Using Trial Sequential Analysis for estimating the sample sizes of further trials: example using smoking cessation intervention

**DOI:** 10.1186/s12874-020-01169-7

**Published:** 2020-11-30

**Authors:** Ravinder Claire, Christian Gluud, Ivan Berlin, Tim Coleman, Jo Leonardi-Bee

**Affiliations:** 1 Division of Primary Care, University of Nottingham, Nottingham, NG7 2RD UK; 2 Copenhagen Trial Unit, Centre for Clinical Intervention Research, Department 7812, Rigshospitalet, Copenhagen University Hospital, Copenhagen, Denmark; 3 Département de pharmacologie, Hôpital Pitié-Salpêtrière, Paris, France; 4 Centre Universitaire de Médecine Générale et Santé publique, Centre Hospitalier Universitaire Vaudois, Lausanne, Switzerland; 5 Division of Epidemiology and Public Health, University of Nottingham, Nottingham, NG5 1PB UK

**Keywords:** Meta-analysis, Trial sequential analysis methods, Trial Sequential Analysis software, Sample size, Information size, Smoking, Pregnancy, Randomised clinical trial, Pilot trial, Feasibility trial

## Abstract

**Background:**

Assessing benefits and harms of health interventions is resource-intensive and often requires feasibility and pilot trials followed by adequately powered randomised clinical trials. Data from feasibility and pilot trials are used to inform the design and sample size of the adequately powered randomised clinical trials. When a randomised clinical trial is conducted, results from feasibility and pilot trials may be disregarded in terms of benefits and harms.

**Methods:**

We describe using feasibility and pilot trial data in the Trial Sequential Analysis software to estimate the required sample size for one or more trials investigating a behavioural smoking cessation intervention. We show how data from a new, planned trial can be combined with data from the earlier trials using trial sequential analysis methods to assess the intervention’s effects.

**Results:**

We provide a worked example to illustrate how we successfully used the Trial Sequential Analysis software to arrive at a sensible sample size for a new randomised clinical trial and use it in the argumentation for research funds for the trial.

**Conclusions:**

Trial Sequential Analysis can utilise data from feasibility and pilot trials as well as other trials, to estimate a sample size for one or more, similarly designed, future randomised clinical trials. As this method uses available data, estimated sample sizes may be smaller than they would have been using conventional sample size estimation methods.

## Background

Demonstrating that health interventions work requires substantial resources. Often feasibility and pilot randomised clinical trials (RCTs) are conducted before larger-scale randomised clinical trials are designed to determine benefits and harms [1–3]. Feasibility trials are used to ascertain information such as intervention acceptability, feasibility of intervention delivery, and recruitment likelihood to help design more decisive RCTs [1]. A pilot trial is a smaller version of a large-scale RCT, and is used to test whether the main components of the trial, such as recruitment, randomisation, treatment, and follow-up assessments can all work together [1]. Moreover, their data can be used to inform sample sizes for large-scale RCTs [2, 3].

Trial sequential analysis is a methodology that can be used in systematic reviews and meta-analyses to control random errors, and to assess whether further trials need to be conducted [4, 5]. Trial sequential analysis as a method can be performed using the Trial Sequential Analysis software, which is freely available alongside its user manual online at The Copenhagen Trial Unit website [6]. Here we employ Trial Sequential Analysis and combine data from feasibility and pilot RCTs testing a text message-based smoking cessation intervention for pregnant women (‘MiQuit’) [7, 8] to estimate the sample size that one or more future RCTs would need to recruit, to provide a more decisive answer regarding the effect of the intervention. We also show how data from the new, planned trial or trials can be combined with data from earlier trials using Trial Sequential Analysis to assess the intervention’s benefits and harms. Using Trial Sequential Analysis sample size estimation methods maximises use of available trial data and consequently, the new RCT or trials may become smaller than they would have been using conventional sample size estimation methods.

### Conventional meta-analysis

Meta-analyses often influence future research; when planning future trials, investigators frequently use meta-analysis to provide an accurate summary of an intervention’s likely effect. If all available RCTs are included, systematic reviews with meta-analyses are considered the best available evidence, because power and precision of the estimated intervention effect is the best one can get [9, 10]. However, this does not necessarily mean that the available evidence is either sufficient or strong. Conventional meta-analysis methods do not consider the amount of the available evidence in relation to the required sample size [11–13]. The reliability of a statistically significant intervention effect generated by meta-analysis is often overvalued, particularly where sparse data (number of events and participants) or repetitive analyses (type I errors) are employed [6, 10, 14, 15]. In other situations, intervention effects that are not statistically significant are often interpreted as showing that the intervention has no effect, and it is assumed that no more evidence is required (type II errors) [16, 17].

In conventional meta-analysis, there is no way to differentiate between an underpowered meta-analysis and a true finding of an intervention being ‘ineffective’. However, it is imperative that a conclusion as to whether an intervention is truly ineffective or truly effective is made as soon as possible after trials are completed, in order to guide investigators’ decisions as to whether further trials could be informative or not [6]. Trial sequential analysis is a methodology that can overcome this issue by distinguishing whether meta-analyses provide evidence for either beneficial or harmful intervention effects, lack of effect (futility), or insufficient evidence for evaluation of the intervention effect [6, 18].

## Methods

### Trial sequential analysis

Meta-analyses aim to discover the benefit or harm of an intervention as early and as reliably as possible. As a result, they tend to be updated when new trials are published [19]. When intervention evaluation has just begun and only few, smaller trials are available, meta-analyses may be conducted on sparse amounts of data and are at high risk of random type I and type II errors [20]. As meta-analyses are updated they are subjected to repeated significance testing, which increases the risk of type I errors [21]. When there are few data available, the Trial Sequential Analysis software resolves these issues by having stringent thresholds for assessing statistical significance, using monitoring boundaries. Monitoring boundaries also take into account the volume of significance testing which has been undertaken through adjusting the thresholds that are used to define whether or not results are considered statistically significant [6].

Trial Sequential Analysis is also able to assess when an intervention has an effect smaller than what would be considered clinically minimally important [6]. Futility boundaries, originally developed for interim analysis in RCTs, can be estimated and used to provide a threshold below which an intervention would be considered to have no clinically important effect [6]. Thus, performing further trials is considered futile as the intervention does not possess the postulated clinically minimally important effect [6].

In Trial Sequential Analysis, when neither the monitoring boundaries nor the futility boundaries are crossed, further information is usually required. Trial Sequential Analysis can also inform how much more information is required to get a conclusive answer regarding the effect of the intervention versus its comparator – this is called the distance between the accrued information and the required information.

#### Required information size

For RCTs, an estimation of the required sample size is performed to ensure the number of participants included is enough to detect or reject a minimum clinically important effect size [17]. For binary outcomes, such as death, the sample size estimation is based on the expected proportion of deaths in the control group, the expected relative risk reduction of the intervention, and the selected maximum risks of both type I and type II errors [18]. Similarly, for meta-analyses to produce adequately powered findings regarding intervention efficacy, sufficient numbers of participants need to be included. This number is referred to as the ‘required information size’ (or ‘optimal information size’ or ‘meta-analytic sample size’) [22, 23]. The meta-analytic required information size can be estimated using similar parameters as those used in sample size estimation for a single trial if one uses a fixed-effect model. If one intends to use a random-effects model, then one needs to consider adjusting for any between-study heterogeneity measured by inconsistency (I^2^) or diversity (D^2^) [18]. Inconsistency is the test statistic for heterogeneity usually used in meta-analysis, and diversity characterises the proportion of between trial variation in any meta-analysis relative to the total model variance of the included trials [24]. Diversity is equal to inconsistency or larger [24]. Heterogeneity between studies is likely to be observed in meta-analyses due to the magnitude of the intervention effect varying when used in different study populations, in studies with different methodological characteristics, or due to variations in the intervention itself [13]. Thus, sample size estimations need to be increased to allow for this between-trial heterogeneity [18].

In the Trial Sequential Analysis software, trials are chronologically ordered, and interim analyses are conducted as each trial is added using summary data from each trial. In a trial sequential analysis where the ‘required information size’ has not been reached, the threshold for statistical significance is inflated to account for sparse data and multiple testing of the interim analyses using monitoring boundaries; thus, the 95% confidence interval is not providing coverage of the real uncertainty and the cut-off for determining statistical significance is below the usual nominal figure of 0.05 [18]. Furthermore, the Trial Sequential Analysis software provides adjusted confidence intervals if the ‘required information size’ has not been reached, which we refer to as Trial Sequential Analysis-adjusted confidence intervals [18]. Technical details regarding how monitoring boundaries, information size, and Trial Sequential Analysis-adjusted confidence intervals are calculated can be found elsewhere [6, 18]. Other statistical software, such as STATA and packages in R, could potentially be programmed to perform trial sequential analysis however to our knowledge these have only been performed on hazard ratios for time-to-event data [25, 26].

In the worked examples below, we show how the Trial Sequential Analysis software can be used to estimate the sample size required for one or more new trials to add further data to a meta-analysis to provide more firm evidence for an intervention either having or not having the postulated effect.

## Results

In this section, we provide an example of how Trial Sequential Analysis successfully used data from feasibility and pilot RCTs that tested MiQuit, a text-message, self-help smoking cessation intervention for pregnant women, to justify research funds to undertake a third, more adequately powered RCT.

### Previous MiQuit trials

Smoking during pregnancy increases the risk of miscarriage, stillbirth, low birth-weight, premature birth, perinatal morbidity and mortality, sudden infant death, as well as adverse infant behavioural outcomes [27, 28]. Pregnancy is a life event which motivates cessation attempts amongst smokers and over 50% of pregnant women who smoke attempt to quit during this time [29], consequently pregnancy is an opportune moment to offer smoking cessation support. Text message, self-help support, smoking cessation programmes developed for non-pregnant smokers are effective, but such programmes are inappropriate for use during pregnancy [30–32]. To address the lack of acceptable self-help, support cessation programmes for pregnant smokers in the UK, MiQuit was developed [7]. MiQuit delivers individually-tailored text messages to pregnant smokers, with the aim of encouraging them to stop smoking [7]. Further details on MiQuit can be found elsewhere [7].

A MiQuit feasibility RCT was conducted, including 207 women. Biochemically-validated, 7-day point prevalence cessation at 12 weeks post randomisation (~ 6 months gestation) was 12.5% in the experimental MiQuit group, compared with 7.8% in the control group (odds ratio (OR) 1.68, 95% confidence interval (CI) 0.66 to 4.31) [7]. Although the trial was small, and the cessation period brief, the trial provided an estimate suggesting that MiQuit could have a positive impact in addition to routine care.

The feasibility RCT lead to minor changes to the intervention, before a pilot RCT was conducted to investigate the feasibility of undertaking a fully-powered multi-centre RCT in UK National Health Service (NHS) settings [8]. The pilot MiQuit RCT recruited 407 pregnant women that smoke, which had largely similar baseline characteristics to those in the feasibility RCT. The self-reported abstinence from 4 weeks post-randomisation until late pregnancy follow-up (approximately 36 weeks gestation) biochemically validated at follow-up was 5.4% in the experimental MiQuit group versus 2.0% in the control group (OR 2.70, 95% CI 0.93 to 9.35) [8]. This trial also suggested a beneficial effect of MiQuit.

As MiQuit is a cheap intervention and can be disseminated widely, it was anticipated that even a 1 to 2% absolute effect on smoking cessation in pregnancy could be clinically important and cost effective [8]. The results from the feasibility and pilot trials suggested that an impact of this size was attainable; however, an adequately powered RCT would still be needed to determine whether MiQuit is effective and guide future routine clinical practise.

### Conventional meta-analysis

The conventional way to determine if an intervention is effective or not is to use the naïve alpha of 5% and the naïve 95% confidence interval [10]. Since both the feasibility and pilot trials used almost the same design as was planned to be used in the new RCT, they can be considered as pilots and it would be appropriate to meta-analyse these trials’ findings together. Using a random-effects model, a traditional meta-analysis of pilot and feasibility studies’ data found, that women randomised to MiQuit were more than twice as likely to be abstinent in their pregnancy (pooled OR 2.26, 95% CI 1.04 to 4.93; I^2^ = 0%, *p* = 0.041). This result seems to be significant according to conventional assessment (*p* < 0.05). However, this result should be interpreted with caution because, as described above, findings from meta-analyses based on only two small RCTs can produce spurious findings due to type I error [11, 12, 22] (please see below).

In the next sections, we use conventional sample size estimation methods to estimate the sample size for an RCT which, on its own would have enough power to show whether MiQuit might be effective, using a plausible treatment effect estimate derived from the conventional meta-analysis above. We also calculate a second sample size estimate for one or more further RCTs, which when pooled with data from feasibility and pilot trials using Trial Sequential Analysis methods, would be similarly decisive.

### Conventional sample size estimation

As the pilot trial [8] was considered at lower risk of bias compared to the feasibility trial [7], a traditional sample size calculation using smoking cessation rate estimates derived from the pilot trial suggests a new trial would require a total sample size of 1292 participants. This estimate has 90% power (10% type II error) and 5% significance (2-sided test; type I error) to detect a 3.4% absolute difference in prolonged abstinence from smoking from 4 weeks after enrolment until 36 weeks gestation between the MiQuit and control groups (5.4% versus 2.0%) [8].

### Trial sequential analysis

**Fig. 1:**
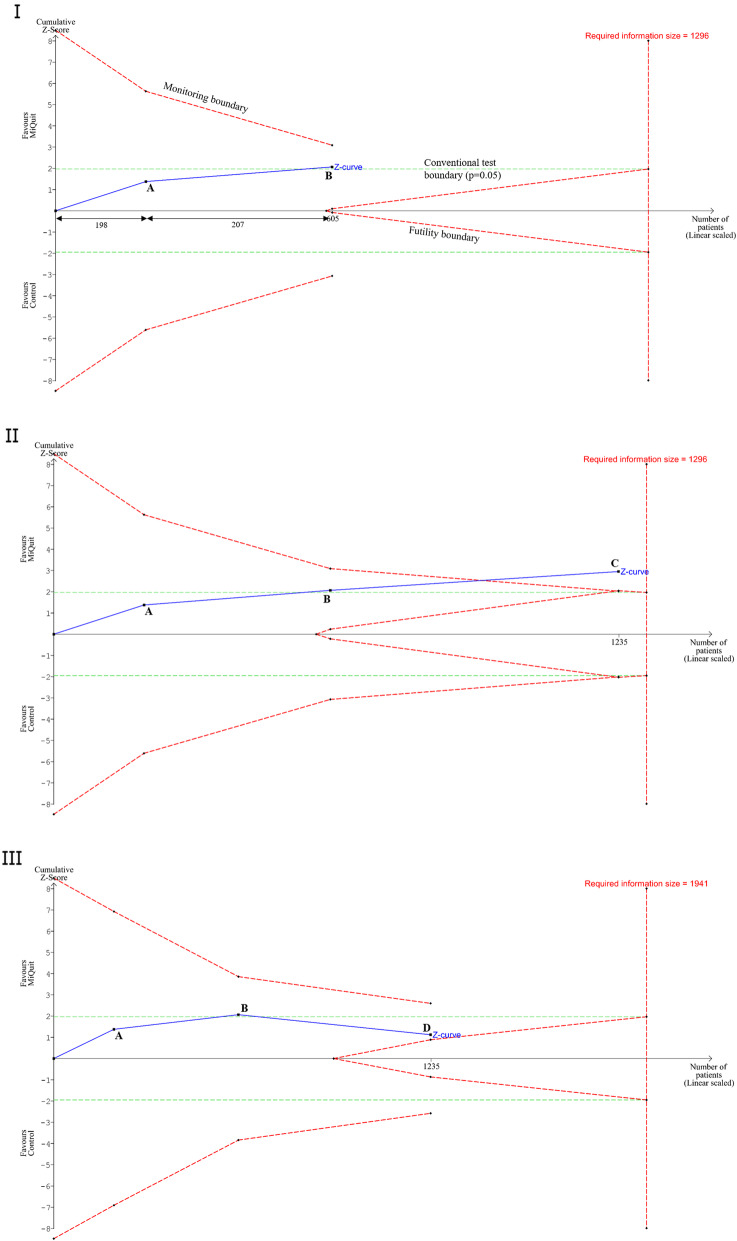
**I.** Trial Sequential Analysis output of both MiQuit trials using 90% power, 5% significance, to detect a 3.4% absolute difference. Diversity = 0%. Points A and B on the z-curve represent each trial added to the sequential analysis. A – Feasibility trial [7], B – Pilot trial [8]. Monitoring boundaries in Fig. 1 use the Lan-DeMets O’Brien-Fleming alpha-spending approach. **II.** Point **C** represents a theoretical trial with a sample size of 630 women, where an absolute difference of 3.17% was observed, in favour of the MiQuit group versus the control group. Diversity = 0%. **III.** Point D represents a theoretical trial with a sample size of 630 women, with an absolute difference of − 0.63% in favour of the control group. Diversity = 33%

Figure 1 I illustrates a Trial Sequential Analysis incorporating findings from the MiQuit feasibility (A) [7] and pilot (B) [8] trials. In this Trial Sequential Analysis output, the x-axis represents the number of participants and marked on this are the numbers of participants recruited to each trial. The y-axis represents the z-score, where a positive z-score favours the MiQuit intervention and a negative z-score favours the control

The z-score is the test that helps you decide whether to accept or reject the null hypothesis. Very high positive or very low negative z-scores are associated with very small *p*-values. The critical z-score values when using a 95% confidence level, which are known as the ‘conventional test boundaries’, are − 1.96 and + 1.96 and these relate to a two-sided *p*-value of 0.05. If the z-score is between − 1.96 and + 1.96, the *p*-value will be larger than 0.05, and the null hypothesis of no difference between intervention groups is accepted. The z-curve represents the cumulative z-score as each RCT is added to the analysis. In Fig. 1.I, when trial B is added to the analysis, the z-curve crosses the conventional test boundary (*p* = 0.05). This is consistent with the results from the conventional meta-analysis for MiQuit, where we found *p* = 0.041.

The required information size is represented by the vertical red line in Fig. 1. The required information size was estimated using the same variables as used for the conventional sample size estimation above (90% power, 5% significance, to detect a 3.4% absolute difference) [8]; although this estimate could take into account observed heterogeneity, there was none in this meta-analysis (I^2^ = 0% and D^2^ = 0). Consequently, the estimated required information size of 1296 participants is only slightly different to that using conventional sample size estimation due to rounding errors. The estimate would be larger if heterogeneity were present.

As the cumulative z-curve does not cross the upper trial sequential monitoring boundary for benefit, this Trial Sequential Analysis shows that further information is required before any firm conclusion can be reached about MiQuit efficacy. Although the conventional meta-analysis suggested, with borderline significance, that pregnant women randomised to MiQuit were more than twice as likely to be abstinent from smoking in late pregnancy, the Trial Sequential Analysis software shows that this finding is not sufficiently robust. The Trial Sequential Analysis-adjusted confidence intervals for cessation using MiQuit (pooled OR 2.26, Trial Sequential Analysis-adjusted CI 0.66 to 7.70), are much wider than those of the conventional meta-analysis (pooled OR 2.26, 95% CI 1.04 to 4.93).

Without Trial Sequential Analysis having been undertaken, an interpretation of the conventional meta-analysis would have been that MiQuit is effective. However, Trial Sequential Analysis indicates that one cannot be secure in this interpretation and further trial data should be collected to eliminate the possibility that this is a false positive result, which can occur early in intervention evaluation when small trials are undertaken.

### Calculating sample size for a third MiQuit RCT

Trial Sequential Analysis has demonstrated that further RCT data are required before a firm conclusion about MiQuit efficacy can be determined. As the initial two trials were sufficiently similar to be combined in Trial Sequential Analysis, we will now demonstrate how Trial Sequential Analysis can be used to estimate the sample size for (a) further trial(s) – data from which, when combined with the previous two trials in the Trial Sequential Analysis software, would be expected to provide a more decisive answer regarding MiQuit efficacy. We will also demonstrate how exemplar theoretical findings from future trials which are both in favour and against MiQuit having a positive effect would impact the Trial Sequential Analysis result.

#### Trial sequential analysis sample size estimation

Estimates derived from the Trial Sequential Analysis found the required information size as 1296 participants. From the feasibility and pilot studies, 605 women have already been recruited and randomised; therefore, the required sample size for further RCTs can be estimated as the difference between the required information size minus the number of women already recruited into the previous trials; thus a sample size of 691 women (346 per intervention group) would be needed, assuming a 1:1 ratio.

Figure 1 II shows the Trial Sequential Analysis output after adding a theoretical third trial (C) with a sample size of 630 women (315 per trial group), where an absolute difference of 3.17% was observed in favour of the MiQuit group versus the control group. The Trial Sequential Analysis clearly shows the cumulative z-curve line crossing the upper trial sequential monitoring boundary which indicates MiQuit being effective. As the trial sequential monitoring boundary has been crossed, the Trial Sequential Analysis z-curve does not need to reach the required information size of 1296. In the present scenario, we can firmly conclude that MiQuit is effective for smoking cessation compared with control (provided that all trials are valid and not influenced by systematic errors (bias) or other errors)

When a theoretical third trial (D) with a negative outcome is included in the Trial Sequential Analysis (Fig. 1.III), we observe a different output. Here, the third trial of sample size 630 was intentionally given a negative outcome (absolute difference of − 0.63% in favour of control). Here we observe the z-curve drop below the conventional test boundary, and in a meta-analysis we would have concluded that MiQuit was not effective. However, in the Trial Sequential Analysis, the futility boundary is not crossed, so we are unable to decisively say that MiQuit is not as effective as control for smoking cessation. Due to the diversity, the required information size has increased to 1941, meaning future trials will need a further 706 participants.

#### A conservative approach to sample size estimation using trial sequential analysis

**Fig. 2:**
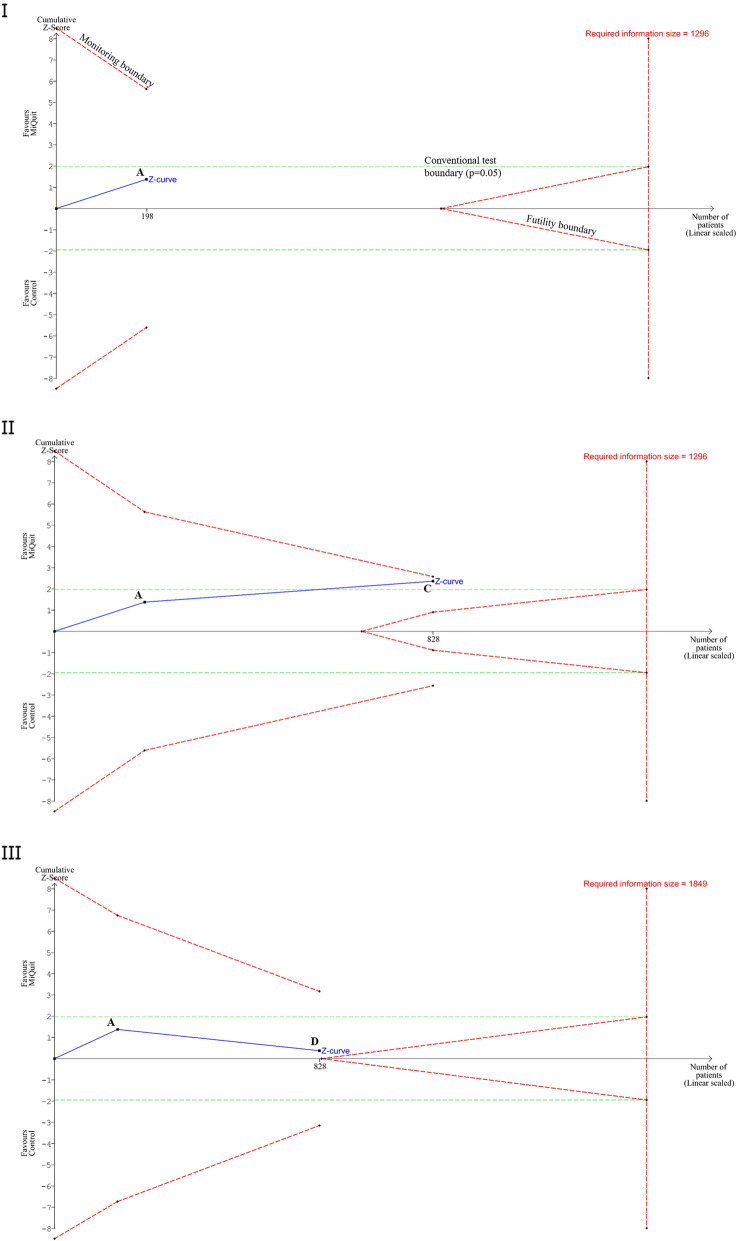
**I.** Trial Sequential Analysis output of the MiQuit feasibility trial with the pilot trial removed, using 90% power, 5% significance, to detect a 3.4% absolute difference. Diversity = 0%. Point A on the z-curve represents the feasibility trial. Monitoring boundaries in Fig. 1 use the Lan-DeMets O’Brien-Fleming alpha-spending approach. **II.** Point C represents a theoretical trial with a sample size of 630 women, where an absolute difference of 3.17% was observed, in favour of the MiQuit group, between the experimental versus the control group. Diversity = 0%. **III.** Point D represents a theoretical trial with a sample size of 630 women, with an absolute difference of − 0.63% in favour of the control group. Diversity = 30%

In the above example, the required information size was derived using the smoking cessation effect from the pilot trial [8]. Therefore, it can be contested whether data from the pilot trial should be included in subsequent Trial Sequential Analysis. Consequently, one could exclude the data from the pilot trial from the Trial Sequential Analysis and re-estimate the total number required (Fig. 2. I). Using this approach, to provide a conclusive result, either a single trial of 1098 participants (549 per intervention group, assuming a 1:1 ratio) or multiple trials cumulating to a total of 1098 participants, would be needed. This figure, although conservative, is still less than the estimate from the conventional sample size calculation.

Figure 2 II and 2.III also show the Trial Sequential Analysis outputs if theoretical trials C and D were included in the analyses. In both situations further information is needed, despite the z-curve coming close to the upper trial sequential monitoring boundary in Fig. 2.II and the futility boundary in Fig. 2. III

### Sensitivity analysis

The modelled scenario, in which there is no heterogeneity between trials in a meta-analysis is rare; in most situations where the described approach is used, some heterogeneity between studies is to be expected. Trial Sequential Analysis provides 95% confidence intervals for heterogeneity (D^2^) within meta-analyses. One way to fully allow for heterogeneity is to perform a sensitivity analysis using the upper 95% confidence interval for the between-trial heterogeneity variance estimate. This would increase the required information size. In our example, the program could not calculate the 95% confidence interval surrounding the D^2^ of 0% as there were less than three included studies. In this case it is possible to input an estimate for heterogeneity into the Trial Sequential Analysis software.

## Discussion

The above example demonstrates how Trial Sequential Analysis can be used to determine the required sample size for one or more additional RCTs to make a meta-analysis more conclusive. This sample size would be considered underpowered in comparison to a traditional RCT sample size calculation. By using Trial Sequential Analysis in such a way, future trials could be planned using significantly fewer resources and with less cost than trials planned using traditional sample size calculations.

In the worked example, data from the pilot trial were used in the Trial Sequential Analysis to estimate the required information size. Ignoring that the same data is being used twice (for the estimation and for the meta-analysis) could mean that the estimate generated is not sufficiently conservative. Thus, we present a modification which attempts to overcome this issue. This approach increases the difference between required information size minus the accrued information by the sample size of the trial used in the estimation.

It is important to note that in the example, the meta-analysis of the existing two MiQuit trials quantified heterogeneity as 0%, indicating no heterogeneity. However, it is unlikely that this will be the case for meta-analyses of other interventions aimed at changing addictive behaviours [33, 34]; therefore, trial sequential analysis methods have been developed to account for this [22]. In Trial Sequential Analysis, estimated information size and monitoring boundaries, vary with the level of heterogeneity in the meta-analysis, the greater the level of heterogeneity, the larger the sample size and the wider the monitoring boundaries needed to reach firm conclusions about the effectiveness of the intervention. This is because the required information size is calculated relative to the measure of heterogeneity, the fraction of the accrued information size and the point estimate [18].

Sometimes trial design is adapted once a study has begun; for example, one or more intervention arms may be dropped and the sample size re-calculated. The method demonstrated in this manuscript is different as it involves using aggregated data in trial planning prior to a study commencing; however, the statistical techniques are analogous to those used in interim trial analysis.

In the examples presented, odds ratios were also used instead of relative risk, as the feasibility study was powered using an odds ratio from a meta-analysis investigating mobile phone interventions for smoking cessation in the general population [7]. Moreover, the quit rates are relatively low, so there is very little difference between the odds ratio and relative risk. In other trial sequential analyses, it may be advisable to use relative risks instead of odds ratios, to avoid overestimates. Additionally, it may be inappropriate to use the odds ratio used to power the feasibility trial to estimate sample sizes for future MiQuit trials since data now exists from the feasibility and pilot trials. In our example, the stipulated intervention effect was derived from the pilot trial (‘internal data’), and it may be argued that such adaptive data should not be used in meta-analysis [35].

Kulinskaya and Wood argued that in an underpowered meta-analysis, not only is it necessary to assess the gap from the accrued information size to the required information size (i.e. the number of additional participants you need to randomise), but also the number of trials that should be conducted to randomise this number of participants [36]. Using multiple trials to reach the required information size may be beneficial in meta-analyses where heterogeneity occurs [36]. Smaller trials have more imprecise estimates of intervention effects; hence heterogeneity is reduced in the meta-analysis of such trials. However, setting up more than one trial can be more expensive and may not be realistic in practice.

Recently, the Cochrane Collaboration evaluated and updated their guidance on using sequential approaches in meta-analysis in their reviews [5, 10, 37]. The Cochrane Handbook authors concluded that sequential methods should not be used in primary analyses or to draw conclusions, but could be used as secondary analyses in reviews if they are prospectively planned and the assumptions underlying the design are clearly justified [5, 10]. In their guidance, the evidence synthesis group state that authors’ interpretations of evidence should be based on estimated magnitude of effect of an intervention and its uncertainty rather than drawing binary conclusions, and decisions should not be influenced by plans for future updates of meta-analyses [10]. These criticisms of sequential approaches in meta-analyses apply to the traditional use of Trial Sequential Analysis, whereas our paper demonstrates an alternative use of the method.

Another reason given by The Cochrane Handbook authors against using sequential methods as a primary analysis in reviews, is the argument that a meta-analyst does not have any control over designing trials that are eligible for meta-analysis [10]. It would therefore be impossible to construct a set of stopping rules [10]. In our example, the opposite is the case. Both the feasibility and pilot trials were conducted by the same group of investigators, and any future trials would have a consideration for the desired properties of a stopping rule.

Finally, The Cochrane Handbook authors also highlight that there are methodological limitations to sequential methods when heterogeneity is present [10]. In the example described in this paper, heterogeneity was not detected, possibly due to the lack of sufficient power to detect a moderate level. However, we do discuss how the presence of heterogeneity can be overcome in Trial Sequential Analysis by performing a sensitivity analysis.

## Conclusions

In conclusion, Trial Sequential Analysis is a freely available software that can utilise data from feasibility and pilot trials as well as other trials, in order to estimate a sample size for one or more future RCTs, to provide an adequately powered conclusion regarding an intervention’s benefits and harms. This simple use of expensively collected trial data could be usefully exploited by researchers evaluating other interventions.

## Data Availability

Trial Sequential Analysis software, user manual and further information regarding the mathematics behind the method are available at http://www.ctu.dk/tsa/ for free. All data generated or analysed during this study are included in the following published articles: Naughton F, Prevost AT, Gilbert H, Sutton S. Randomized controlled trial evaluation of a tailored leaflet and SMS text message self-help intervention for pregnant smokers (MiQuit). Nicotine & Tobacco Research. 2012;14 (5):569–77. Naughton F, Cooper S, Foster K, Emery J, Leonardi-Bee J, Sutton S, et al. Large multi-centre pilot randomized controlled trial testing a low-cost, tailored, self-help smoking cessation text message intervention for pregnant smokers (MiQuit). Addiction. 2017;112 (7):1238–49.

## Abbreviations

CI: Confidence interval
NHS: National Health Service
OR: Odds ratio
RCT: Randomised clinical trial
